# British Gynæcological Society

**Published:** 1889-06

**Authors:** 


### BRITISH GYNÆCOLOGICAL SOCIETY.

### CONSTRICTED SEA-TANGLE TENT.

Dr. Bantock showed this, as gynæcologists denied the existence of constriction of the internal os. The patient was a young married lady who had had incision of the cervix performed in 1886 by severe dysmenorrhœa; about a week later a glass-stem was introduced, but had to be removed speedily. Five months later she had an attack of what was called pelvic peritonitis. Eight months ago he found the uterus very tender and retroverted. The left ovary was enlarged and prolapsed. The question of removal of the appendages, even if suggested, would not have been entertained. He could only adapt a pessary and enjoin rest. This treatment conferred relief from the general symptoms, the ovarian tenderness, but not from the dysmenorrhœa. Thinking it advisable to dilate the uterus, he found the obstruction would not yield to graduated bougies, and wishing to explore the uterus, he introduced a tangle tent. At the end of twenty-four hours it would not move, in forty-eight it was removed with difficulty, and a glance at the tent shown must convince the most skeptical as to the constriction. Dysmenorrhœa due to a uterine condition was far more frequently to be found at the internal than at the external os. No local or constitutional disturbance followed.

MASSAGE.

The discussion on Dr. Macnaughton Jones' paper was resumed. — Dr. Bantock feared that in a great measure massage had degenerated into a system of quackery. In some of the cases reported the patients had been subjected to many operations under a wrong diagnosis, and he had seen several similar ones—for example, a case of simple retroversion which needed only a simple support for the cure, and a second in which cystic disease of the ovaries was the real cause, and the removal of them effected complete relief. — Dr. Edis, Dr. Fancourt Barnes, and Dr. Fenton referred to the injurious effects of this method of treatment, more especially in cases of pelvic inflammation, which were frequently not recognized.—Dr. Grigg reported several cases in which massage has been most beneficial in diseases of the liver, stomach, and in muscular affections; he knew nothing of it as applied to the pelvic viscera, but referred to some serious mistakes in its application.—The President said he did not share in the strong objections that had been taken to uterine massage. If, by such treatment (and he must say it had not been clearly described), a practitioner could cure his patients, it was right to adopt it. Were not the very same objections urged against the use of the speculum originally? If massage had been so abused (which he did not deny), it was probably because it had been attended with success.—Dr. Jones, in reply, said he had never used massage in internal pelvic conditions, because he was afraid of the danger of such a course. He regretted the discussion had dealt with the abuse rather than the use of what was, in suitable cases, a most successful method of dealing with diseases that were otherwise exceedingly prolonged in duration and very intractable.

